# The Research Data Centre of the German Federal Employment Agency at the Institute for Employment Research (RDC-IAB) – Linked Microdata for Labour Market Research

**DOI:** 10.23889/ijpds.v4i2.1141

**Published:** 2019-11-20

**Authors:** M Antoni, A Schmucker

**Affiliations:** 1 Institute for Employment Research (IAB) of the German Federal Employment Agency (BA) Regensburger Strasse 104 D-90478 Nuremberg

## Abstract

**Background and data portfolio:**

Since 2004, the Research Data Centre of the German Federal Employment Agency at the Institute for Employment Research (RDC-IAB) has been offering comprehensive individual data on employees, unemployed persons, job seekers and participants in active labour market policy programmes for scientific labour market research. For this purpose, data from employer notifications and from different administrative processes in the labour market administration are linked. These administrative data are also combined with survey data. In addition, linked employer-employee data allow simultaneous analyses of the supply and demand sides of the labour market.

**Data linkage:**

The data can be linked using unique identifiers, such as social insurance numbers, client numbers from local employment agencies, or establishment numbers. Since the foundation of the German Record Linkage Center (GRLC) in 2011, the RDC-IAB also applies methods for linking with non-unique and error-prone linkage identifiers like names, addresses and birth dates.

**Data access:**

German data protection law classifies the data offered by the RDC-IAB as highly sensitive and strictly regulates their use by external researchers. The RDC-IAB has therefore established various data access modes. Although data can be transferred directly to research institutions in anonymised form, this procedure is generally not effective for linked data, as the loss of information due to the necessary anonymisation would be too great. For this reason, the RDC-IAB focuses on the access modes on-site use and remote data execution. In cooperation with other data centres, RDC-IAB has therefore established on-site data access at currently 16 locations worldwide.

## Background

Over the last decades, the need for extensive data in the social sciences has increased considerably. At the same time, public data producers were already collecting extensive amounts of data, which, however, were hardly available for research. In addition, demand for evidence-based policy consulting in Germany increased. For example, a legal requirement of scientific evaluations was part of the labour market reforms in the years 2003 to 2005 (the so-called ‘Hartz-reforms’) in order to better assess the effectiveness of labour market policy [1].

In the year 1999, the German Federal Ministry of Education and Research recognised the need for action and instructed the “Commission to improve the statistical infrastructure in cooperation with the scientific community and official statistics” to draw up proposals to make data from registers and statistics more accessible for scientific research [2]. The commission explored the current state of the data infrastructure and recommended the establishment of research data centres (RDC) by public data producers.

## Approach

### Governance, legislation and management

#### Institutional setup

As a consequence, the German Federal Employment Agency (BA), among others, was requested to establish an RDC. For legal reasons, only the Institute for Employment Research (IAB) as its independent research unit1The IAB is a special office of the BA with a statutory mandate to conduct labour market research. The institute is independent, i.e. freedom of research and publication is guaranteed. was permitted to store the BA’s administrative data permanently. Moreover, as the IAB had been preparing, documenting and using these data for labour market research for several years, comprehensive experience with these tasks was already available at the IAB. For these reasons, the BA established the RDC-IAB as a department of the IAB in 2004. After initial funding of three years by the German Federal Ministry of Education and Research, the RDC-IAB was subsequently financed by the BA as a permanent institution. In addition, various projects are financed through the acquisition of third party funding [1]. Access to RDC-IAB’s standardised datasets, on the other hand, has always been cost-free for researchers and their institutions.

The main tasks of RDC-IAB are the preparation, standardisation and documentation of data available to the IAB. Furthermore, it enables secure access to microdata for non-commercial labour market research and offers advisory service on data selection and handling [1]. The RDC-IAB provides samples from the BA’s administrative data, survey data as well as datasets that combine different data sources. As the RDC-IAB is a research unit instead of an archive, its staff members do their own research using IAB’s data. Moreover, the RDC-IAB frequently opens up new data sources and develops new data products during third party funded projects, often in collaboration with external researchers. Hence, RDC-IAB staff members have a deep knowledge about the data they provide, which enables them to advise users about the data’s analytic potential, scope and validity.

The RDC-IAB has started providing linked datasets as early as 2005. While early linkages only relied on unique identifiers such as social security or establishment numbers, the need for capabilities in indirectly linking datasets using non-unique identifiers quickly became evident. In collaboration with Prof. Rainer Schnell (University of Duisburg-Essen), the RDC-IAB therefore established the German Record Linkage Center (GRLC) as a cooperation project in 2011. Initially, the German Research Foundation (DFG) funded the GRLC2The DFG grants SCHN 586/17-2 and BE 3172/1-2 ended in 2016 and 2015, respectively.. After that funding ended, the GRLC was continued as an informal collaboration between the University of Duisburg-Essen and the RDC-IAB. While the former has been very successful in its research on, among other things, privacy preserving record linkage, the RDC-IAB has been providing fee-based record linkage services [3]3See http://record-linkage.de for more details and for current publications on both research and linkage projects..

#### Development

The RDC-IAB has steadily expanded its portfolio of datasets, working tools and services. To guide such developments, RDC-IAB has always taken the requirements of the research community into account, for example by monitoring demand for its datasets and access types or by conducting user surveys at irregular intervals and publishing their results [4-7]. In these user surveys, the RDC-IAB asks about users’ satisfaction with its services, the preferred access types and the data required. In addition, the RDC-IAB remains in close contact with its data users at conferences, its own data user workshops and through research collaboration.

The number of available data products increased from five in 2005 to more than 20 in 2019. New data products often emerge from the staff’s own research. For example, the RDC-IAB has recently started offering the “Inventor biography data linked to administrative data of the IAB” [DOI: 10.5164/IAB.INV-BIO-ADIAB8014.de.en.v1, 8], which were initially linked, processed and documented for a cooperation project between an employee of the RDC-IAB and the Max Planck Institute for Innovation and Competition.

To develop new data products, the RDC-IAB also raises third-party funds competitively in order to ensure that the needs of the scientific community are met. For example, the RDC-IAB is involved in the DFG Priority Program “The German Labour Market in a Globalised World: Challenges through Trade, Technology, and Demographics”. As part of this project, the RDC-IAB supplies custom shaped data to researchers of this program. These data are specifically designed to suit the research questions of the specific projects. Furthermore, the RDC-IAB aims to incorporate innovations achieved in this program into its standardised data products to make them available for other researchers as well. As a first result, the RDC-IAB has added the “FDZ Sample of the Administrative Wage and Labor Market Flow Panel” [FDZ-AWFP, DOI: 10.5164/IAB.FDZ-AWFP7614.en.v1, 9] to its portfolio of standardised data products in 2019. The FDZ-AWFP was developed in close cooperation with researchers in the DFG Priority Program.

There has also been considerable developments in data access modes. RDC-IAB focuses on the improvement of on-site access, e.g., by following the RDC-in-RDC approach [10]. While on-site data access was initially only possible in Nuremberg, users currently can access the data at 16 locations in Germany, France, England, Canada and the USA.

The RDC-IAB is also working on making remote data access more user-friendly, e.g., by allowing remote job execution through the web-based software “JoSuA” (Job Submission Application) since 2015. A detailed description can be found in [11]. The latest user survey showed that users regard JoSuA as an improvement, but consider its user-friendliness to be in need of improvement [4]. The RDC-IAB will therefore finance the further development of JoSuA.

Data quality has always been of great importance to the RDC-IAB, which is why it conducts methodological research on data quality, e.g., by using linked data in validation studies [12, 13] or in analyses of survey nonresponse [14]. Data users also contribute to improving data quality, often by publishing data-related results in the FDZ-Methodenreport series. If possible, the RDC-IAB uses these results to implement improvements in new or updated datasets. For example, a solution for resolving inconsistent or missing education information developed by Fitzenberger, Osikominu [15] was implemented in the administrative data [16].

Researchers of the RDC-IAB or of other departments of the IAB also conduct research to evaluate the success of data linkages and the quality of linked data. For instance, as informed consent is necessary for most data linkages in Germany, many analyses focus on linkage consent bias [17, 18].

#### Legislative basis for data collection and privacy

As administrative data provided by RDC-IAB comprise mandatorily collected information, they are subject to a very high level of protection.4Although participation in surveys conducted by the IAB is voluntary, the resulting survey data are subject to the same confidentiality requirements as social security data. The legal basis for the collection of data on dependent employment is the German Data and Transmission Act (Datenerfassungs- und -übermittlungsverordnung - DEÜV) and German Social Code (Sozialgesetzbuch - SGB) Book IV. Additionally, SGB Books II and III apply for the collection of data on internal procedures of employment agencies [1]. Article 282, subparagraph 5 of SGB Book III permits the IAB to use the administrative data for research purposes. Furthermore, there are several legal regulations for the dissemination of anonymised research data to external researchers (article 282, subparagraph 7, SGB Book III and article 75, SGB Book X). Furthermore, the regulations of the Federal Data Protection Act (Bundesdatenschutzgesetz - BDSG) and the General Data Protection Regulation (GDPR) of the EU apply. To facilitate compliance with these regulations, the RDC-IAB receives advice on data protection issues from the IAB’s legal department and from the data protection officer of the BA. The RDC-IAB is also subject to the Federal Ministry of Labour and Social Affairs as supervisory authority.

### Population setting

**Figure 1: Sources and development of RDC-IAB’s standard data products:**
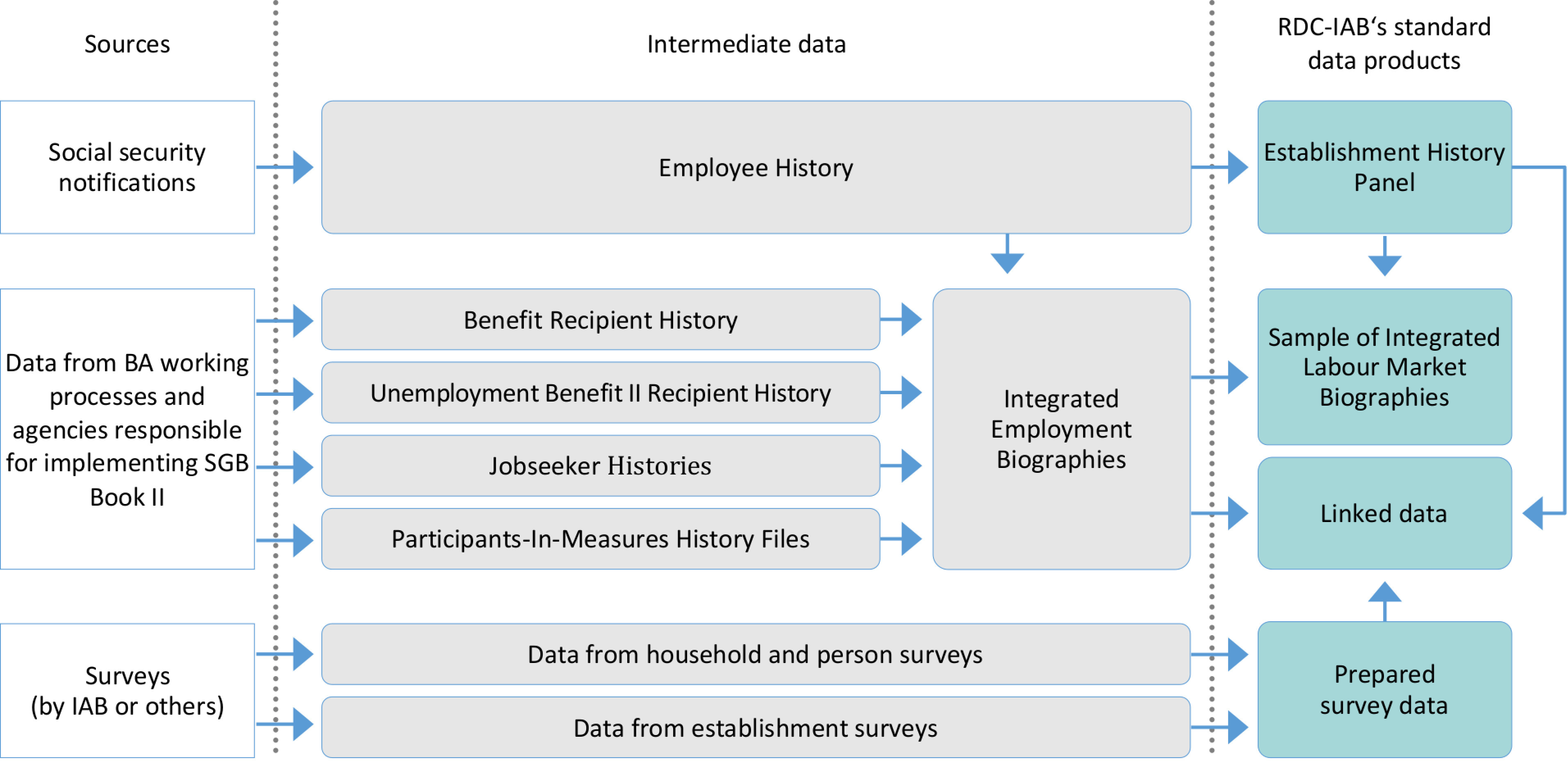


As shown in Figure 1, the RDC-IAB receives data from different sources. Its administrative data originate from two sources: First, notifications of employers to the social security system, which contain information on every employment relationship subject to the German social security system. Second, the process-generated data from the internal procedures of the BA, which provide information on unemployed, job seekers, benefit recipients and participants in active labour market policy programs.

### Data sources and data portfolio

The IAB department “Data and IT-Management” combines all notifications for the social security system to create the “Employee History”. In the second step, the first data linkage takes place as data from different sources have to be combined. These datasets include unique identifiers such as the social security number or client numbers of unemployed or benefit recipients. In collaboration with the statistics and the controlling departments of the BA, the IAB has developed a method to create a unique person identifier to be able to link data across all of these sources. By doing so, the department “Data and IT-Management” generates the “Integrated Employment Biographies” [IEB, 19], which contain information on a daily basis and, at least for some of the sources, cover all relevant persons since 1975 for West Germany and 1992 for East Germany.

Furthermore, the IAB conducts own surveys on persons, households and establishments to complement its administrative data. For some of these surveys, the IAB collaborates closely with other institutions (see Section “Survey Data”).

The RDC-IAB only provides access to standardized datasets that offer a broad analysis potential and are therefore suitable for a large number of projects. The following subsections will focus mainly on central data products that receive regular updates.5See https://fdz.iab.de/en/FDZ_Overview_of_Data.aspx for an overview on all currently available datasets.

#### Administrative data

The “Sample of Integrated Labour Market Biographies” (SIAB) is a two percent random sample of the “Integrated Employment Biographies”. The current version (DOI: 10.5164/IAB.SIAB7517.de.en.v1) provides longitudinal data on about 1.9 million individuals over the period 1975-2017 [20]. RDC-IAB updates the SIAB roughly every two years. Figure 1 shows the data sources that contribute to the SIAB.

The “Establishment History Panel” (DOI: 10.5164/IAB.BH P7517.de.en.v1) is a longitudinal dataset composed of yearly cross-sections with June 30^th^ as the reference date for the period 1975-2017. The data contain a 50 percent random sample of all establishments in Germany with at least one dependent employee (between 640,000 and 1.5 million establishments per year). The data comprise information about the industry, the location, the number of workers in different employee groups as well as means and medians of wages. Additional datasets provide information about worker flows and about foundations and closures of establishments [21, 22]. The data are updated annually and are linked to all administrative or linked data products.

### Survey data

Given the large number of surveys the IAB conducts or collaborates in, this subsection focuses on survey data that are also available as part of linked datasets. Established by the IAB in 2007, the annual “Panel Labour Market and Social Security” (DOI: 10.5164/IAB.PASS-SUF0618.de.en.v1) surveys about 8,000 to 12,000 households per wave with between 12,000 and 19,000 interviewed persons. The panel allows analyses on the dynamic of the receipt of unemployment benefit and the effects of receipt of social benefits on the economic and social situation of the affected households and individuals [23]. RDC-IAB makes the data of the waves available within one year after the end of each field phase.

The “IAB-SOEP Migration Sample” (DOI: 10.5684/soep.iab-soep-mig.2017) is a household survey conducted jointly by the IAB and the German “Socio-Economic Panel” at the German Institute for Economic Research. The dataset offers insights into aspects such as the structure of immigration and the labour market integration of migrants. The first five survey waves were carried out between 2013 and 2017, with about 3,400 to 5,000 persons (about 1,900 to 2,700 households) taking part in each of them (24). RDC-IAB releases updates of this dataset annually.

The “IAB Establishment Panel” (DOI: 10.5164/IAB.IABBP 9317.de.en.v11) is an annual representative establishment survey on various topics, e.g., on the determinants of labour demand. It has been running since 1993 and contains information on up to 16,000 establishments per wave [25]. RDC-IAB makes the data of new waves available within one year after the end of the field phase.

### Linked data

The RDC-IAB started providing access to linked data directly after its foundation. By now, the RDC-IAB offers ten distinct linked datasets, or even more if one counts the different variants that some of these datasets have. This subsection describes two linked datasets with very distinct features. Table 1 provides an overview of all linked datasets currently available.

**Table 1: Overview of linked data products of the RDC-IAB:** 

Dataset name	Sources	Observational units	Linkage method	Reference

ALWA survey data linked to administrative data of the IAB (ALWA-ADIAB)	administrative and survey data	persons, establishments	probabilistic via name, address, birth date, sex	[29]
Biographical data of selected insurance agencies in Germany (BASiD)	administrative data of BA and German Pension Fund	persons, establishments	directly via unique social security number	[30]
IAB-SOEP Migration Sample linked to administrative data of the IAB (IAB-SOEP-MIG-ADIAB)	administrative and survey data	persons, households, establishments	directly via unique person identifier, probabilistic linkage via name, address, birth date	[24]
Inventor biography data linked to administrative data of the IAB (INV-BIO ADIAB)	administrative and publicly available data	persons, establishments, patents	probabilistic via name, address	[8]
IZA/IAB Linked Evaluation Dataset 1993-2010 (LED)	administrative and survey data	persons	directly via unique person identifier	[31]
Linked Personnel Panel (LPP) survey data linked to administrative data of the IAB (LPP-ADIAB)	administrative and survey data	persons, establishments	directly via unique establishment and person identifiers	[32]
Linked-Employer-Employee-Data of the IAB (LIAB)	administrative and survey data	persons, establishments	directly via unique establishment and person identifiers	[26]
National Educational Panel Study (NEPS), Starting Cohort 6 (SC6) survey data linked to administrative data of the IAB (NEPS-SC6-ADIAB)	administrative and survey data	persons, establishments	probabilistic via name, address, birth date, sex	[33]
Panel Labour Market and Social Security survey data linked to administrative data of the IAB (PASS-ADIAB)	administrative and survey data	persons, households, establishments	directly via unique person identifier, probabilistic linkage via name, address, birth date, sex	[34]
WeLL survey data linked to administrative data of the IAB (WeLL-ADIAB)	administrative and survey data	persons, establishments	directly via unique establishment and person identifiers	[35]

The first among those linked datasets was the “Linked Employer-Employee Data of the IAB” (LIAB), which still is one of the most demanded datasets of the RDC-IAB. For this dataset, the IAB Establishment Panel survey data are directly linked to administrative establishment data by using the BA’s unique establishment number. This is possible because the survey’s sample is drawn from IAB’s administrative establishment data. The same establishment number is used to also link the “Integrated Employment Biographies” data of all workers employed at the surveyed establishments. The LIAB therefore allows simultaneous analyses of the supply and demand sides of the German labour market [26]. RDC-IAB provides different models of LIAB and updates them roughly every two years.

One of the most recent linked datasets is called “National Educational Panel Study (NEPS), Starting Cohort 6 (SC6) survey data linked to administrative data of the IAB” (NEPS-SC6-ADIAB, DOI: 10.5164/IAB.NEPS-SC6-ADIAB7515.de.en.v1). The special features of NEPS-SC6-ADIAB are, first, that the survey data on their own [27] are prepared, documented and provided by the RDC of the Leibniz Institute for Educational Trajectories [LIfBi, 28]. Second, the LIfBi provides funds to the RDC-IAB, which in turn provides access to the combined dataset via on-site use and remote data execution. The collaborating RDCs plan updates of NEPS-SC6-ADIAB roughly every two years.

### Consent model

Informed and active (opt-in) consent is required when linking survey data to IAB’s administrative data. In general, there is a requirement of written consent, although the GDPR and German legislation leave room for well-founded exceptions to collect verbal consent, e.g., in case the written form would negatively impact data quality or could introduce selection bias. However, no consent is required when publicly available data are considered, e.g., when linking patent register data to inventors and their employers in IAB’s administrative data.

No individual consent is required for the linkage of the data across different data sources within the BA. This is also not necessary for linking with data of the German Pension Fund, since the same legal basis applies for the collection of the data. However, informed consent is usually required for linking with administrative data from other data producers (e.g., the German Federal Statistical Office, health insurance companies).

### Data linkage keys

As Table 1 shows, the IAB performs record linkage based on both unique identifiers such as social security or establishment numbers and on non-unique identifiers like names, addresses and birth dates. Preference is given to the former, although such unique identifiers are almost only available when the sample for a given survey is drawn from the address databases of the BA in the first place. Depending on the target population of a survey, it can be more suitable to draw a general population sample from municipal registration offices instead of from the labour force population represented in IAB data. In such cases, a subsequent linkage usually has to rely on the non-unique identifiers mentioned above.

Before the comparison step, the RDC-IAB always performs an extensive pre-processing of such identifiers. To facilitate this, the GRLC has developed a set programs both in R and in Stata that deal with issues that commonly arise in German name and address data.

### Data linkage

Unless a unique identifier is available, the RDC-IAB performs a stepwise record linkage on non-unique and error-prone identifiers. The linkage workflow usually involves a number of deterministic linkage steps in which exact agreement on linkage identifiers is checked. The strictness is decreased iteratively, e.g., by removing the house number from the linkage key. These steps are always followed by a distance-based or probabilistic comparison for the remaining unlinked cases, again in an iterative process that allows for some variation in the elements of the linkage key.

To increase efficiency in these steps, the RDC-IAB applies traditional blocking, mostly on exact agreement of the whole or part of the postcode, on the birth year, on sex or on some combination of these blocking identifiers. The RDC-IAB commonly uses different blocking variables or combinations thereof iteratively to reduce false-negative matches and increase the linkage success rate.

Like the pre-processing, the deterministic comparison steps are usually performed in R or Stata. Depending on the software environment the workflow was started with, the distance-based or probabilistic linkage steps are either performed using the R-package PPRL6For more details, see https://cran.r-project.org/package=PPRL. or the Java-based software Merge ToolBox [MTB, 36]. Both were developed by the GRLC unit based at the University of Duisburg-Essen.

### Data access

#### Privacy by design: the data disclosure portfolio

To protect the privacy of persons and establishments included in the data of the RDC-IAB, access is governed by strict data protection laws. Hence, the RDC-IAB has developed a data disclosure portfolio to provide these sensitive data to external users [37]. The RDC-IAB follows the approach by Lane, Heus [38], which is based on principles of data providers worldwide. They define four scopes of action: technical, organisational, statistical and legal measures. In practice, the RDC-IAB uses the following protective measures:

**Checking the eligibility of data usage:** According to legal regulations, only research projects in the field of labour market research are permitted. Applicants for data access must also show that their research is not feasible with other less restricted data. Only independent scientific research institutions are eligible as applicants.

**Provisions on data access:** Data use agreements are concluded. In addition, data users assure to comply with the RDC-IAB’s data protection regulations and terms of use.

**Anonymisation of data:** We distinguish between the anonymisation of data before they are used and the disclosure control of the results afterwards. For the anonymisation of data, first, population samples are drawn. Second, identifiers like names, addresses and social security numbers are deleted. Third, sensitive variables are removed. This still results in restricted data, which must not be transmitted directly to users. For the transmission, the data are subjected to further anonymisation steps. Here, the RDC-IAB follows the recommendations of Müller, Blien [39]. In general, only non-perturbative methods (e.g., global, top and bottom coding, deletion of digits of hierarchical classifications or sensitive variables) are applied [40], as users prefer information reduction to data-perturbing interventions [41]. However, these methods lead to considerable loss of information, especially with linked data. Therefore, the RDC-IAB uses alternative data access modes to make these restricted data available for research as well.

**Secure computing environment and input/output control:** Restricted data may only be stored and processed in the secure computing environment at the RDC-IAB. As far as the analysis results are concerned, users can view them within the secure environment, but results must undergo a disclosure control before they can be released. The aim of the disclosure control is to ensure that the released results are absolutely anonymous and that no conclusions can be drawn about individual persons or establishments. A detailed description of the procedure can be found in [37].

**Figure 2: Relationship of degree of anonymity and data access type:**
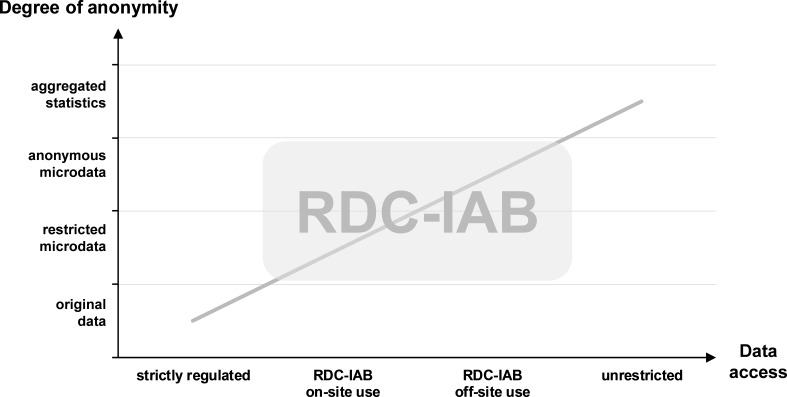


Figure 2 shows the relationship between the degree of anonymity and the data access types at the RDC-IAB. The RDC-IAB offers three different ways of data access: the dissemination of anonymized scientific use files (SUFs, off-site use), on-site access and remote data execution.

#### Application process

In order to obtain access to data, applicants must first complete a request form, which is available online in German and English. The application also requires a short project description to check for feasibility and legal requirements. One of these requirements is that only projects in the field of labour market research are permitted. Applicants for data access must also show that their research is not feasible with other, less restricted data. Furthermore, it must be proven that the applicant is an independent scientific research institution. If the transfer of a SUF is requested, an additional data security concept must be submitted, which describes the technical and organisational measures for data processing within the research institution. If all requirements are fulfilled, the RDC-IAB approves the project and a time-limited data use agreement is concluded. Ethics approval is not required. The agreement sets out guidelines for the users and sanctions in the event of violations.

#### Data use

The data are made available in accordance with the contractually agreed data access modes:

- SUFs may be downloaded from an exchange server via a secure internet connection by the user.
- To be able to use restricted data on-site or via remote data execution, each research project receives its own directory with the contractually specified data. Different users within a project receive separate accounts and passwords.

The use of the data is limited to the duration of the use agreement. After the contract end date, all extracts and copies of the SUF at the research institution have to be deleted. In the case of restricted data, access by the user to the project directory will be blocked after the end of the contract and the contents of the directory will be archived for ten years.

### Architecture and information technology

SUFs are only provided in the Stata data format. However, after downloading the data, users can freely choose the software they run their analyses with, as long as they are able to import the original data.

While working on-site in the secure environment of the RDC-IAB, where USB ports are blocked, no internet connection is available and users cannot install their own software, the range of software products is more limited. Most users rely on Stata, but it is also possible to work with R or, with a strongly limited number of licenses, Matlab. However, the guidelines of the RDC-IAB require users to only use R or Matlab for tasks that cannot be performed with Stata.

Submitting remote data execution jobs via JoSuA is currently restricted to Stata. Users that need to perform remote data execution with software other than Stata are able to submit their syntax via email.

### Noteworthy outputs by data users

Since the foundation of the RDC-IAB, data users have published hundreds of research articles. A comprehensive overview of publications based on data of the RDC-IAB can be found in this literature database: https://fdz.iab.de/en/FDZ_Publications/FDZ_Literature_Database.aspx.

The following two are the latest publications based on linked data (see Table 1 for more details on the datasets mentioned here): Liepmann [42] used BASiD to analyse the impact of a negative labour demand shock on fertility in East Germany after the fall of the Berlin Wall in 1989. While birth rates in West Germany remained relatively stable after 1989, the fertility in East Germany declined. The paper shows that women that were more severely affected by the demand shock had relatively more children than less severely affected women.

Reichelt and Abraham [43] used ALWA-ADIAB to investigate the influences of and returns (i.e., wage increase or staying employed) to occupational and regional job mobility and argue that these two mobility types (change of occupation or move to another region) act as substitutes when employees aim to improve their wage or avoid unemployment.

In addition, key players in policy consulting in Germany use the data of the RDC-IAB, such as the Minimum Wage Commission, which periodically adjusts the minimum wage level in Germany, or the German Council of Economic Experts, the most important independent committee of experts for economic policy issues. The research based on our data is also used for comprehensive reports to German federal ministries. For example, results on atypical forms of employment based on the dataset NEPS-SC6-ADIAB were included in the “German Federal Government’s 5th Report on Poverty and Wealth” [44, 45]. Furthermore, the “Second Gender Equality Report of the German Government” contains research results on the gender pay gap drawn from analyses of RDC-IAB data users [46].

## Discussion

The RDC-IAB continues to regularly update and expand its portfolio, in particular with regard to linked data. A promising avenue to achieve this is an increased collaboration with other data producers. One such extension is already underway: together with the RDC of the LIfBi, the RDC-IAB will provide access to three additional NEPS starting cohorts linked with administrative data of the IAB.

Access to linked data is generally possible, but the increased richness of data also increases the risk of de-anonymisation. Established ways of access to the single data sources may not be suitable for their combination. Therefore, the RDC-IAB plans to expand the possibilities of remote data processing. If data protection regulations permit, we strive to implement real-time remote access. Meanwhile, the possibilities for on-site use will also be expanded. Additional locations in Europe are in the planning stage.

In addition, the RDC-IAB is working on improving the documentation of its data. To this end, it introduced Digital Object Identifiers (DOI) for data products and publications in 2018. Moreover, a metadata database and an associated web information system based on the DDI standard are being developed on behalf of the RDC-IAB to facilitate both the maintenance and the search for metadata.
